# Patterns of HIV viremia and viral suppression before diagnosis of non-AIDS-defining cancers in HIV-infected individuals

**DOI:** 10.1186/s13027-015-0033-x

**Published:** 2015-11-03

**Authors:** David J. Riedel, Anne F. Rositch, Robert R. Redfield

**Affiliations:** Institute of Human Virology and Division of Infectious Diseases, University of Maryland School of Medicine, Baltimore, MD USA; Department of Epidemiology, Johns Hopkins Bloomberg School of Public Health, Baltimore, MD USA; Institute of Human Virology and Division of Infectious Diseases, University of Maryland School of Medicine, Program in Oncology, University of Maryland Marlene and Stewart Greenebaum Cancer Center, 725 W. Lombard St., N552, Baltimore, MD 21201 USA

**Keywords:** HIV, AIDS, Viremia, Non-AIDS-defining cancer, Anal cancer, Hodgkin lymphoma

## Abstract

**Background:**

The association between HIV viremia and non-AIDS-defining cancers (NADCs) is not well characterized. Viremia may contribute directly or indirectly to cancer development and may have a differential impact on various cancer types. Our objective was to characterize patterns of HIV viremia in a retrospective, urban, clinical cohort (N = 320) of patients diagnosed with NADCs.

**Findings:**

The most common NADC’s were lung (n = 60), prostate (n = 47), oropharyngeal (n = 32), liver (n = 29), and anal cancer (n = 20) and Hodgkin lymphoma (n = 18). In the year before cancer diagnosis, 66 % of all patients were virally suppressed. Patients with oropharyngeal (70 %) and prostate cancer (78 %) had a higher proportion of visits with suppressed viral loads. Patients diagnosed with anal cancer and Hodgkin lymphoma were infrequently virally suppressed and more frequently had viral loads ≥5 log_10_ copies/ml in the ten years prior to cancer diagnosis.

**Conclusions:**

In this cohort of HIV-infected patients diagnosed with NADCs, there were important differences in the patterns and levels of viremia between the different NADCs in the ten years prior to cancer diagnosis. Patients with anal cancer and Hodgkin lymphoma had the highest proportion of high level viremia in the ten years before cancer and the lowest frequency of viral load suppression at cancer diagnosis.

## Introduction

Cancer risk is markedly increased in HIV-infected individuals compared to the general population, and it has become a frequent cause of morbidity and mortality in this population. Multiple factors contribute to the increased risk of both AIDS-defining cancers (ADCs) and non-AIDS-defining cancers (NADCs), including viremia, immune deficiency, oncogenic virus co-infection (e.g. hepatitis B/C), behavioral carcinogen exposures (e.g. alcohol and tobacco), aging, and possibly antiretroviral therapy (ART) [1–8].

HIV viremia may contribute directly to development of ADCs, but for NADCs, the impact is not as well characterized and may be indirect [2, 7, 9]. Viremia as a risk factor for malignancies has been measured in various ways, including as duration of high-level circulating virus (i.e., viral load (VL) ≥5 log_10_) [10], peak VL [11], current or time-lagged nonsuppressed VL [2], and cumulative viremia (i.e. approximate area under the curve) [3, 9], and appears to play a fundamental role in the development of non-Hodgkin lymphoma (NHL) [2, 3, 10]. However, the actual mechanism by which circulating virus contributes to lymphomagenesis is not yet known, and the association and oncogenic mechanisms between HIV viremia and NADCs is understudied.

The purpose of this retrospective cohort study was to characterize patterns of HIV viremia and viral suppression in the ten years preceding NADC diagnosis and to compare these factors among the six most common NADCs (Hodgkin lymphoma and oropharyngeal, anal, liver, lung, and prostate cancer) and to ADCs in a diverse, urban clinic population.

## Methods

Medical records of all HIV-infected individuals diagnosed with cancer in the University of Maryland Medical System and Baltimore Veterans Affairs Medical Center from January 2000 to December 2011 were reviewed. Data abstraction methods have been previously described [12]. Cancer diagnoses were confirmed by pathology and clinician reports and were categorized as ADCs (Kaposi sarcoma, invasive cervical cancer, and NHL) or NADCs (all other cancers except the three ADCs). The most common NADCs were further divided into infection-related (anal and liver cancer and Hodgkin lymphoma), infection-unrelated (lung and prostate cancer), and mixed (oropharyngeal cancer) [13]. Although oropharyngeal cancers include a mixture of human papillomavirus (HPV)-related and HPV-unrelated subtypes [14], data on HPV DNA tissue testing or specific anatomical subsites were not available. Institutional Review Boards at each site approved the protocol.

All patients with ≥1 HIV VL measurement in the ten years prior to cancer diagnosis were included in this analysis. Measurements below the level of detection were assumed to be 1 log_10_. HIV suppression was defined as an HIV RNA VL ≤400 copies/ml (assays over time had varying detection limits). HIV duration was time from HIV diagnosis to cancer diagnosis.

Characteristics of patients are described for NADCs and ADCs overall and for the six most common NADCs, along with the proportion of clinical visits before cancer diagnosis with viral suppression. Categorical variables were compared using chi square, and continuous variables were compared using Wilcoxon rank sum or student’s *t* test. Levels of viremia (log_10_) in the 10 years preceding diagnosis are plotted with a fitted linear trend line to examine differences across time. Data analysis was performed using SAS version 9.3 (SAS Institute Inc., Cary, NC, USA).

## Results

There were 320 patients diagnosed with NADCs and 105 with ADCs. Characteristics of the patients are shown in Table 1. The median age at NADC diagnosis was 54 (IQR: 48-59) years, and 68 % of NADCs were in patients older than 50 years compared to 76 % of ADCs in patients 50 years or younger. The median number of VL measurements per NADC patient was 12 (IQR: 3-23). The most common NADC’s in this population were lung (n = 60), prostate (n = 47), oropharyngeal (n = 32), liver (n = 29), and anal cancer (n = 20) and Hodgkin lymphoma (n = 18) (Appendix).

**Table 1:** Demographic and clinical characteristics of patients diagnosed with ADCs and NADCs

	All ADCs	All NADCs	*P* value*	Infection-related NADC			Infection-unrelated NADC		Mixed^a^
	(n = 105)	(n = 320)							
				Hodgkin	Anal	Liver	Lung	Prostate	Oral
				(n = 18)	(n = 20)	(n = 29)	(n = 60)	(n = 47)	(n = 32)
Characteristics	N (%) or Median (IQR)			N (%) or Median (IQR)					
Year of diagnosis									
2000-2005	39 (37 %)	90 (28 %)		7 (39 %)	4 (20 %)	5 (17 %)	19 (32 %)	8 (17 %)	9 (28 %)
2006-2011	66 (63 %)	230 (72 %)	0.08	11 (61 %)	16 (80 %)	24 (83 %)	41 (68 %)	39 (83 %)	23 (72 %)
Age (years)									
≤50 years	80 (76 %)	101 (32 %)		13 (72 %)	15 (75 %)	5 (17 %)	17 (28 %)	1 (2 %)	10 (31 %)
>50 years	25 (24 %	219 (68 %)	<0.01	5 (28 %)	5 (25 %)	24 (83 %)	43 (72 %)	46 (98 %)	22 (69 %)
Male	81 (77 %)	255 (80 %)	0.58	14 (78 %)	19 (95 %)	29 (100 %)	52 (87 %)	47 (100 %)	23 (72 %)
Black race	89 (85 %)	280 (88 %)	0.04	17 (94 %)	18 (90 %)	24 (83 %)	55 (92 %)	44 (94 %)	31 (97 %)
Smoking									
Current/Former	75 (71 %)	284 (89 %)		15 (83 %)	18 (90 %)	27 (93 %)	59 (98 %)	43 (91 %)	30 (94 %)
Never	30 (29 %)	36 (11 %)	<0.01	3 (17 %)	2 (10 %)	2 (7 %)	1 (2 %)	4 (9 %)	2 (6 %)
Alcohol									
Current/Former	37 (35 %)	188 (59 %)		9 (50 %)	6 (30 %)	26 (90 %)	40 (67 %)	33 (70 %)	26 (81 %)
Never	68 (65 %)	132 (41 %)	<0.01	9 (50 %)	14 (70 %)	3 (10 %)	20 (33 %)	14 (30 %)	6 (19 %)
Injection drug use									
Current/Former	43 (41 %)	165 (52 %)		7 (39 %)	8 (40 %)	20 (69 %)	32 (53 %)	19 (40 %)	21 (66 %)
Never	62 (59 %)	155 (48 %)	0.06	11 (61 %)	12 (60 %)	9 (31 %)	28 (47 %)	28 (60 %)	11 (34 %)
HIV Transmission									
IDU	38 (36 %)	145 (45 %)		4 22 %)	6 (30 %)	18 (62 %)	28 (47 %)	19 (40 %)	22 (69 %)
Heterosexual	33 (31 %)	104 (33 %)		7 (39 %)	1 (5 %)	5 (17 %)	24 (40 %)	19 (40 %)	9 (28 %)
MSM	29 (28 %)	50 (16 %)		6 (33 %)	11 (55 %)	5 (17 %)	6 (10 %)	5 (11 %)	0 (0 %)
Transfusion	0 (0 %)	3 (1 %)		0 (0 %)	0 (0 %)	0 (0 %)	1 (2 %)	0 (0 %)	0 (0 %)
Unknown	5 (5 %)	18 (6 %)	0.07	1 (6 %)	2 (10 %)	1 (3 %)	1 (2 %)	4 (9 %)	1 (3 %)
Hepatitis C	42 (40 %)	166 (52 %)	0.03	5 (28 %)	9 (45 %)	22 (76 %)	28 (47 %)	27 (57 %)	22 (69 %)
HIV duration (years)^b^	6.4 (1.9-12.2)	11.0 (5.6, 15.3)	<0.01	6.3 (4.1, 14.2)	14.0 (8.4, 16.4)	13.6 (7.0, 15.0)	11.4 (5.6, 15.7)	10.8 (5.3, 14.7)	14.2 (7.8, 19.0)
CD4 cell count^c^									
≤200	37 (38 %)	197 (68 %)		6 (33 %)	9 (53 %)	17 (68 %)	32 (60 %)	36 (80 %)	15 (56 %)
>200	48 (50 %)	65 (22 %)		10 (56 %)	6 (35 %)	5 (20 %)	15 (28 %)	4 (9 %)	8 (30 %)
Unknown	12 (12 %)	28 (10 %)	<0.01	2 (11 %)	2 (12 %)	3 (12 %)	6 (11 %)	5 (11 %)	4 (15 %)
Average no. of HIV RNA VL per patient	6	15	<0.01	10	15	14	13	21	15
Log_10_ VL (copies/ml)^c^	4.5 (2.9-5.1)	1.7 (1.0, 3.9)	<0.01	3.0 (1.0, 4.4)	1.0 (1.0, 2.0)	1.9 (1.0, 4.4)	2.9 (1.0, 4.4)	1.0 (1.0, 2.2)	1.0 (1.0, 3.7)
HIV RNA VL suppression ≤400^c^	23 (34 %)	182 (63 %)	<0.01	9 (50 %)	13 (76 %)	13 (52 %)	25 (47 %)	34 (76 %)	17 (63 %)
ART at diagnosis	34 (32 %)	187 (58 %)	<0.01	6 (33 %)	15 (75 %)	13 (45 %)	31 (52 %)	39 (83 %)	17 (53 %)

*P value is for comparison of ADCs vs. NADCs

^a^Oropharyngeal cancers typically include both human papillomavirus-related and -unrelated cancers

^b^Missing HIV duration data: NADC's (2), Lung (1)

^c^N = 290 NADC's with measurements taken within one year before cancer diagnosis. Missing data by cancer individual cancers: Hodgkin (0), Anal (3), Liver (4), Lung (7), Prostate (2), Oral (5)

*ADCs* AIDS-defining cancers, *ART* antiretroviral therapy, IDU injection drug use, *NADCs* non-AIDS-defining cancers, *VL* viral load

In the year preceding cancer diagnosis, 66 % of all patients with NADCs were virally suppressed compared to only 24 % of patients with ADCs (Table 2). Patients with oropharyngeal (70 %) and prostate cancer (78 %) had higher proportions of visits with suppressed VL in the same time frame, while patients with Hodgkin lymphoma (56 %) and anal cancer (59 %) had lower frequencies of suppression prior to cancer diagnosis. There was significant variability in the proportion of VL suppression at 5-6, 7-8, and 9-10 years before cancer diagnosis (Table 2). Patients with Hodgkin lymphoma had a low proportion of visits with viral suppression before cancer onset (20 % at 3-4 years, 0 % at 5-6 years, 8 % at 7-8 years, and 0 % at 9-10 years). Overall 60 % of all visits in NADC patients in the 10 years before cancer diagnosis had suppressed VL.

**Table 2:** All NADCs and the six most common individual NADCs: percent (N) of visits with viral suppression (HIV RNA viral load ≤400 copies/ml) before cancer onset

Cancer	Within 1 year of onset	Within 2 years of onset	3-4 years prior to onset	5-6 years prior to onset	7-8 years prior to onset	9-10 years prior to onset
All ADCs	24 % (45)	18 % (12)	30 % (45)	41 % (41)	43 % (18)	38 % (11)
All NADCs	66 % (577)	61 % (429)	61 % (681)	56 % (468)	55 % (317)	65 % (220)
Infection related						
Hodgkin	64 % (30)	48 % (23)	20 % (10)	0 % (0)^a^	8 % (1)	0 % (0)^b^
Anal	65 % (42)	53 % (28)	41 % (39)	20 % (11)	53 % (10)	40 % (2)
Liver	66 % (42)	64 % (28)	64 % (47)	68 % (49)	74 % (49)	65 % (26)
Infection unrelated						
Lung	51 % (80)	50 % (66)	60 % (107)	62 % (78)	46 % (33)	44 % (27)
Prostate	79 % (118)	76 % (91)	71 % (154)	56 % (106)	49 % (74)	66 % (52)
Mixed						
Oral	71 % (52)	69 % (43)	74 % (105)	71 % (61)	55 % (35)	50 % (17)

^a^There were 0 visits with viral suppression out of 22

^b^There were 0 visits with viral suppression out of 3

**Fig. 1:**
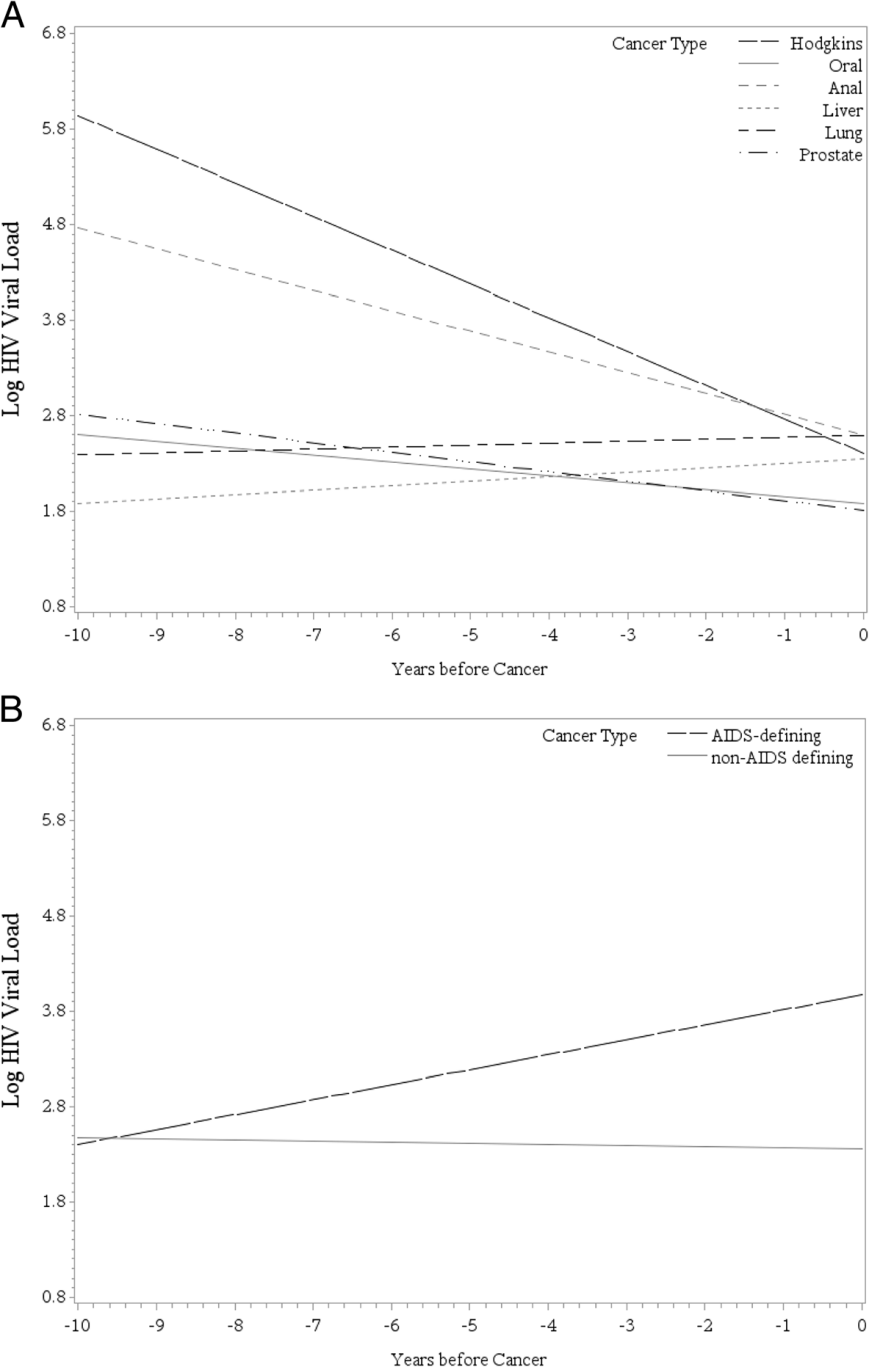
Patterns of log_10_ HIV RNA viral load prior to cancer diagnosis for the six most common NADCs (**a**) and for NADCs versus ADCs (**b**)

The linear pattern of the individual log_10_ VL measurements across time before cancer diagnosis for the six NADCs is shown in Fig. 1a. The slope of viremia curves for both Hodgkin lymphoma and anal cancer were negative, while curves for the other four NADCs were comparatively flat. Figure 1b shows the pattern of VL across time for NADCs compared to ADCs. When further examining the level of VL among all patients with NADCs, only 6.6 % of visits in the ten years before diagnosis had very high VL (e.g. log_10_ VL ≥5). Patients with anal cancer (19.4 %) and Hodgkin lymphoma (17.5 %) had the highest frequency of ≥5 log_10_ VL measurements, while those with prostate (2.7 %), liver (3.6 %), lung (5.1 %), and oropharyngeal (6.9 %) cancers had the fewest.

## Discussion

In this urban cohort of HIV-infected patients diagnosed with cancer, there were important differences in the patterns of viral suppression and levels of viremia between those with ADCs and NADCs and among the different NADCs in the ten years prior to cancer diagnosis. In general, patients with NADCs had a high frequency of VL suppression and rarely had high level viremia prior to cancer diagnosis. Comparing the six most common NADCs, patients with anal cancer and Hodgkin lymphoma had the highest proportion of high level viremia in the ten years before cancer and the lowest frequency of VL suppression at cancer diagnosis. Conversely, patients with prostate cancer had the highest frequency of VL suppression at cancer diagnosis and the lowest frequency of high level viremia prior to cancer diagnosis. These differing patterns of viremia suggest that certain infection-related NADCs (anal cancer and Hodgkin lymphoma) may be more appropriately categorized with ADCs.

Consistent with earlier studies [2, 7], the majority of NADCs in this cohort occurred in patients who were virally suppressed, raising the possibility that other mechanisms associated with HIV pathogenesis may account for the increased incidence of some NADCs. HIV-related immune activation, chronic inflammation, and immunodeficiency likely also play a role in the development of many non-viral NADCs [15]. The high prevalence of certain chronic viral infections (e.g. hepatitis B/C) [4] and behavioral carcinogens (such as smoking) [5] in the HIV-infected population also contribute to the increased NADC risk. Determining the etiologic role and attributable risk fraction of these different factors remains a critical area of future research.

Compared to the four other NADCs studied, both anal cancer and Hodgkin lymphoma had a high frequency of visits with ≥5 log_10_ VL in the ten years preceding cancer diagnosis and were also less frequently virologically suppressed at the time of cancer diagnosis. This finding is consistent with prior work showing that cumulative HIV viremia was associated with development of Hodgkin lymphoma and anal cancer but not hepatocellular cancer [9]. Both anal cancer and Hodgkin lymphoma are etiologically associated with co-infecting oncogenic viruses (HPV and Epstein Barr virus, respectively), and the co-existence of persistent, high level circulating HIV viremia with associated immunosuppression impairs the ability to control and clear these viral co-infections [16]. HIV-infected patients are also more likely to have anal HPV infections, to have multiple, concurrent HPV infections, and to carry high-risk, oncogenic HPV subtypes [17]. These factors likely combine to increase the risk for these particular cancers in the HIV-infected population [13]. The results from this study support previous work suggesting that virally associated NADCs like anal cancer and Hodgkin lymphoma may behave differently than typical non-virally associated NADCs and more like an ADC, with HIV itself as a contributory risk factor. Continuing to categorize cancers in HIV-infected patients into the dichotomy of ADCs and NADCs may no longer be supported by emerging biological and epidemiological data.

A strength of this study is the diverse clinical cohort of urban, HIV-infected patients with a high prevalence of African-Americans, injection drug use and hepatitis C virus co-infection, and smoking, which is underrepresented in the literature. Most prior work is from geographic areas or populations with a high proportion of white race and male-to-male sexual contact as the predominant HIV risk factor [13, 18]. As the study population consists largely of inner city African-Americans of low socioeconomic status, the results may not be generalizable to all HIV-infected patients in other settings. Additionally, the study inferences are limited by the retrospective and clinical nature of the data – VL measures were not taken at standard intervals, although reflecting the true nature of clinically observed patterns of HIV viremia. Patients with certain NADCs had more frequent VL measures than others, possibly implying that viremia could be a surrogate for poor engagement in HIV care and higher rates of high-risk behaviors. We also did not have access to a non-cancer comparison group or comprehensive data on traditional cancer risk factors, limiting the ability to make more direct conclusions about the association of viremia with cancer development and diagnosis. Lastly, differences in ART utilization and efficacy may also have contributed to differences in viremia among the various groups.

In conclusion, we found that patients with NADCs largely had suppressed VL at cancer diagnosis. Similar to ADCs, those with anal cancer and Hodgkin lymphoma were less frequently virally suppressed in the years preceding their cancer diagnosis than patients with prostate, lung, liver, or oropharyngeal cancers. As NADCs continue to increase as a cause of non-AIDS-related morbidity and mortality despite viral suppression, further research should investigate the role of circulating viremia in the pathogenesis of these cancers.

## Appendix

**Table 3:** Number and distribution of non-AIDS-defining cancers

Cancer type	N (%)
Lung	60 (14.1 %)
Prostate	47 (11.1 %)
Oral cavity and oropharynx	32 (7.5 %)
Liver/bile duct	29 (6.8 %)
Anal	20 (4.7 %)
Hodgkin lymphoma	18 (4.2 %)
Non-melanoma skin	16 (3.8 %)
Kidney	14 (3.3 %)
Breast	14 (3.3 %)
Intestine	13 (3.1 %)
Acute myeloid leukemia	7 (1.7 %)
Pancreas	7 (1.7 %)
Penis	7 (1.7 %)
Unknown primary	7 (1.7 %)
Bladder	5 (1.2 %)
Vulvar/vaginal	4 (0.9 %)
Ovary	3 (0.7 %)
Multiple myeloma	2 (0.5 %)
Castleman disease	1 (0.2 %)
Melanoma	1 (0.2 %)
Other leukemia	1 (0.2 %)
Stomach	1 (0.2 %)
Sarcoma	1 (0.2 %)
Testicular	1 (0.2 %)
Thyroid	1 (0.2 %)
Others	8 (1.9 %)

## Abbreviations

ADC: AIDS-defining cancer
ART: Antiretroviral therapy
HIV: Human immunodeficiency virus
HPV: Human papillomavirus
IQR: Interquartile range
NADC: Non-AIDS-defining cancer
NHL: Non-Hodgkin lymphoma
VL: Viral load.
